# A network pharmacology perspective for deciphering potential mechanisms of action of *Solanum nigrum* L. in bladder cancer

**DOI:** 10.1186/s12906-021-03215-3

**Published:** 2021-01-25

**Authors:** Yang Dong, Lin Hao, Kun Fang, Xiao-xiao Han, Hui Yu, Jian-jun Zhang, Long-jun Cai, Tao Fan, Wen-da Zhang, Kun Pang, Wei-ming Ma, Xi-tao Wang, Cong-hui Han

**Affiliations:** 1 Department of Urology, XuZhou Central Hospital Affiliated to Nanjing University of Chinese Medicine, Jiefang South Road, No. 199, Jiangsu Xuzhou, China; 2 Department of Clinical Medicine, Xuzhou Medical University, Xuzhou, China; 3 Xuzhou Clinical Medical College of Integrated Traditional Chinese and Western Medicine Affiliated to Nanjing University of Traditional Chinese Medicine, Xuzhou, China; 4 Center of Reproductive Medicine, Shanghai First Maternity and Infant Hospital, Tongji University School of Medicine, Shanghai, China; 5 Yantai Hospital of Traditional Chinese Medicine, Yantai, China; 6 Department of Urology, Suqian People’s Hospital of Nanjing Drum-Tower Hospital Group, The Affiliated Suqian Hospital of Xuzhou Medical University, Suqian, China; 7 Department of Biotechnology, College of Life Sciences, Jiangsu Normal University, Xuzhou, China

**Keywords:** Bladder cancer, Enrichment analysis, Network pharmacology, *Solanum nigrum* L., Target prediction

## Abstract

**Background:**

*Solanum nigrum* L. decoction has been used as a folklore medicine in China to prevent the postoperative recurrence of bladder cancer (BC). However, there are no previous pharmacological studies on the protective mechanisms of this activity of the plant. Thus, this study aimed to perform a systematic analysis and to predict the potential action mechanisms underlying *S. nigrum* activity in BC based on network pharmacology.

**Methods:**

Based on network pharmacology, the active ingredients of *S. nigrum* and the corresponding targets were identified using the Traditional Chinese Medicines for Systems Pharmacology Database and Analysis Platform database, and BC-related genes were screened using GeneCards and the Online Mendelian Inheritance in Man database. In addition, ingredient-target (I–T) and protein–protein interaction (PPI) networks were constructed using STRING and Cytoscape, Gene Ontology (GO) terms and Kyoto Encyclopedia of Genes and Genomes (KEGG) pathway enrichment analyses were conducted, and then the pathways directly related to BC were integrated manually to reveal the pharmacological mechanism underlying *S. nigrum*-medicated therapeutic effects in BC.

**Results:**

Seven active herbal ingredients from 39 components of *S. nigrum* were identified, which shared 77 common target genes related to BC. I-T network analysis revealed that quercetin was associated with all targets and that *NCOA2* was targeted by four ingredients. Besides, interleukin 6 had the highest degree value in the PPI network, indicating a hub role. A subsequent gene enrichment analysis yielded 86 significant GO terms and 89 significant pathways, implying that *S. nigrum* had therapeutic benefits in BC through multi-pathway effects, including the HIF-1, TNF, P53, MAPK, PI3K/Akt, apoptosis and bladder cancer pathway.

**Conclusions:**

*S. nigrum* may mediate pharmacological effects in BC through multi-target and various signaling pathways. Further validation is required experimentally. Network pharmacology approach provides a predicative novel strategy to reveal the holistic mechanism of action of herbs.

**Supplementary Information:**

The online version contains supplementary material available at 10.1186/s12906-021-03215-3.

## Background

Despite being one of the most common malignancies worldwide, bladder urothelial carcinoma remains refractory, and its incidence and mortality rates have been the highest among all genitourinary tumors for many years in China [1]. Although bladder cancer (BC) is mostly not invasive to muscles at the initial presentation, it regularly recurs, causing fatigue in most patients, and approximately 30% of patients eventually progress to muscle-invasive BC with 5 years [2]. In patients with localized, advanced-stage BC, platinum-based combination chemotherapy continues to be the first line of therapy [3]. Despite the increased use of multimodality therapy with neoadjuvant chemotherapy followed by radical cystectomy or bladder sparing approaches [4–6], the long-term survival rates of patients with muscle-invasive BC have remained largely unchanged for decades [7, 8], and approximately 50% of cases progress to incurable metastatic BC [3, 9]. It has been reported that the use of immunotherapy with checkpoint inhibitors (CPIs) improves the outcomes of patients with metastatic disease [3, 8, 10]. In recent years, five CPIs, including three programmed cell death ligand-1 (PD-L1) inhibitors, namely atezolizumab, avelumab, and durvalumab, and two anti-PD-1 antibodies, nivolumab and pembrolizumab, have been approved by the Food and Drug Administration as systemic therapeutic agents for patients with metastatic and muscle-invasive BCs [3, 10]. However, the efficacy and safety of CPIs in non–muscle-invasive BC are currently under investigation in multiple clinical trials [10]. Although CPIs revolutionized the treatment of BC, only approximately 20% of patients respond to immunotherapy, and the overall prognosis remains dismal [3].

Transurethral resection of bladder tumor followed by intravesical administration with Bacillus Calmette-Guérin or chemotherapy agents such as mitomycin, valrubicin, or gemcitabine is usually associated with a high incidence of local and systemic side effects, such as cystitis-like irritative voiding symptoms, hematuria, skin rash, arthralgia, fever, and influenza-like symptoms, which result in treatment discontinuation in 4–7% of patients [2]. Besides, adjuvant chemotherapy is generally accompanied by significant toxicity, primarily manifesting in the form of anemia, neutropenia, fatigue, nausea, and vomiting [9]. In addition to the toxicity of immune checkpoint inhibitors, the prohibitive costs of such drugs limit their clinical application [2, 10]. Consequently, the development of novel and more effective and economical therapeutic strategies of BC treatment with low or even no toxicity or side effects is required.

Natural plants have been considered as an important source of therapeutic agents for human health for a long time. The beneficial effects of phytochemical drugs on the prevention and treatment of tumors have recently attracted much interest in the field of modern biomedicine. Traditional Chinese medicine (TCM) is composed of derivatives from natural plants, which has a long history of use as remedies for diseases with the focus lying on maintaining the dynamic balance of the entire body to achieve a state of harmony between the human being and nature. *Solanum nigrum* L., a herbal plant indigenous to Southeast Asia, is frequently used as a valuable ingredient in clinical TCM cancer therapy [11]. A previous study has shown that *S. nigrum* leaves contains a compound that can induce autophagy in breast adenocarcinoma cells [12] and apoptosis in hepatoma cells [13]. The mature fruits of *S. nigrum* can inhibit cell growth and, in turn, promote apoptosis in breast cancer cells [14]. By altering the expression of matrix metalloproteinases and inhibiting epithelial–mesenchymal transition, *S. nigrum* extracts effectively inhibits the invasion of prostate cancer cells [15]. In addition, a water extract of *S. nigrum* suppresses melanoma metastasis [16]. Such findings demonstrate that *S. nigrum* can induce antineoplastic activity as a chemopreventive anti-cancer agent. Since a long time, *S. nigrum* decoction has been used as a folklore medicine in China to prevent postoperative BC recurrence; however, its specific mechanism of action has remained unclear. Its active ingredients, cellular targets, and the molecular mechanisms of action in BC are yet to be elucidated.

Network pharmacology, a novel advanced analytical technology, is currently popular owing to its reliability and efficiency in pharmacological research [17]. The key principle of network pharmacology involves applying a network-based approach to construct a multilevel network (drug-gene-path-disease) to explore the therapeutic effects and mechanisms of action of drugs in complex therapies at organizational and molecular levels [18]. Such therapies have the characteristics of wholeness and systematic behavior, which are consistent with the principles of a holistic view and dialectical treatment associated with TCM [19]. Such a high-efficiency research strategy is emerging as the preferred approach for studies investigating the pharmacological mechanisms of TCM herbs and formulas. The therapeutic targets and pathways of such herbs and formulations can subsequently be verified in in vivo and in vitro experiments.

**Fig. 1:**
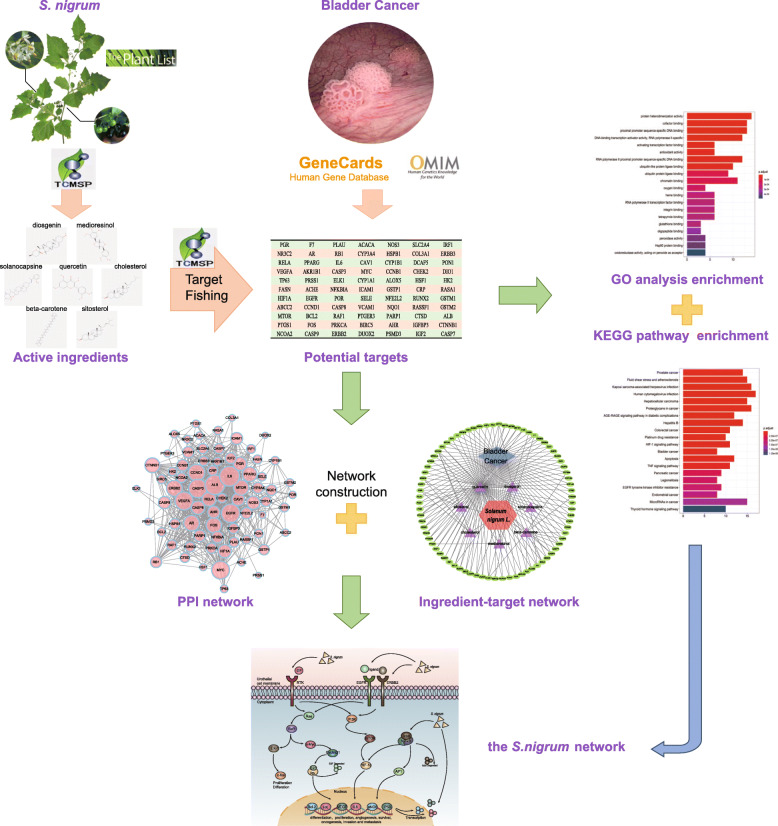
The protocol of the systems pharmacology approach used in the present study

In the present study, we aimed to systematically analyze and explore the active components, specific targets, and the precise molecular mechanisms of *S. nigrum* in BC based on network pharmacology approach. The basic scheme of the pharmacology of the integrated extraction system is illustrated in Fig. 1. We performed a preliminary screening of the active ingredients of *S. nigrum*, and then identified the potential molecular targets of the ingredients and retained those responsible for the occurrence and progression of BC. Subsequently, the interactions among the potential targets were investigated, and Gene Ontology (GO) terms classification and pathway enrichment analysis were further performed. Ultimately, we visualized an integrated “*S. nigrum* pathway” to provide a systematic overview of the potential molecular mechanisms of action of *S. nigrum* activity in BC. The results of the present study might be able to provide a novel approach for an in-depth understanding of the antineoplastic activity of this TCM.

## Methods

### Screening the active ingredients of *S. nigrum*

The herb’s name, “*Solanum nigrum* L.,” was checked in The Plant List (http://www.theplantlist.org/; version 1.1). The major components of *S. nigrum* were identified using the Traditional Chinese Medicines for Systems Pharmacology Database and Analysis Platform (TCMSP; https://tcmspw.com/tcmsp.php; updated on May 31, 2014), which is a well-known Chinese herbal medicine database providing correlations between diseases, drugs, and targets. To explore the potential primary active ingredients, we evaluated each candidate’s druggability based on its corresponding oral bioavailability (OB) and drug likeness (DL) values predicted in TCMSP database. OB refers to the degree and speed of drug absorption, when administered through the mouth, into the circulatory system [20]. DL refers to the similarity between a compound and a known drug in terms of physicochemical properties and structural factors, including solubility, permeability, and stability, which usually allows the pharmacokinetic properties and safety of a compound to be assessed [21]. Generally, higher OB and DL values indicate that the compound will be more valuable for clinical application. As recommended by TCMSP, compounds meeting both the criteria of OB ≥ 30% and DL ≥ 0.18 are perceived to have relatively high affinity, intrinsic activity, and pharmacological properties and can be considered as candidate ingredients for further studies [20–22]. The data of which part of *S. nigrum* the ingredients extracted from was obtained using the Phytochemical Composition Database (http://www.organchem.csdb.cn/scdb/main/plant_introduce.asp).

### Molecular target prediction

Owing to the diversity of *S. nigrum* constituents, the herb can target a variety of proteins; therefore, target prediction is a key step in exploring the molecular mechanism underlying the pharmacological properties of *S. nigrum*. In the present study, target prediction was performed using the TCMSP database, which integrates information from the DrugBank and Herb Ingredients’ Targets (HIT) databases and the Systems Drug Targeting (SysDT) model [22]. DrugBank is a comprehensive knowledgebase that focuses on combining detailed drug data with drug action and target information [23]. Experimentally validated association between herbal compounds and their corresponding targets could be retrieved from the HIT database [24]. As for compounds without validated targets, the SysDT model was used to predict the potential drug-target interaction, which was based on random deep forest and support vector machine [25]. In addition, the UniProt Knowledgebase (UniProtKB; www.uniprot.org/; updated on July 23, 2020) [26] was used to unify the non-canonical description of the identified targets in the “*Homo sapiens*” category, and the normalized molecular targets along with their corresponding gene symbols were finally retrieved.

### Identification of the targets of BC

The genes associated with BC were collected from two sources. One was the GeneCards database, which is recognized for its comprehensive information on all annotated human genes, proteins, and diseases [27]. This database is user-friendly, integrative, and reliable; it aggregates information from 125 different databases, such as the HUGO Gene Nomenclature Committee, National Center for Biotechnology Information, Ensembl, and UniProtKB. Using the GeneCards database (https://www.genecards.org/; version 5.0), information on the related targets was retrieved easily by performing keyword-based searches. The other information resource used was the Online Mendelian Inheritance in Man (OMIM) database (http://www.omim.org/; updated on May 4, 2018), which catalogs current diseases with genetic components and links them to the relevant genes in the human genome [28]. Here, we searched the GeneCards and OMIM databases to identify BC-related targets, with “bladder cancer” as the keyword.

### Ingredient-target (I–T) network construction

Cytoscape is a software platform for complex network analysis and visualization [29]. In the present study, we displayed the interactions between ingredients and targets in a comprehensible manner, by constructing an I–T network using a graph visualization software, Cytoscape (version 3.7.1) (http://www.cytoscape.org/). The layout algorithm (attribute circle layout) was applied. To make the overall visual effect more distinct and easier to understand, Cytoscape allows users to adjust the geometric position of every node and symbol, the colors, and the graphics of the network topology depending on personal preferences. The degree and betweenness centrality are two of the most crucial parameters of the network’s topological structure and are generally used to evaluate the essentiality of each part. These parameters facilitated the identification of the ingredients or targets that played key roles in the action of *S. nigrum* against BC.

### Protein–protein interaction network construction

Internal proteins generally regulate a range of biological functions by interacting to form macromolecular complexes. Thus, exploring the interactions among proteins could provide a reliable theoretical foundation for clarifying the mechanisms of drug action, improving drug efficacy, and preventing adverse reactions. Search Tool for the Retrieval of Interacting Genes/Proteins (STRING) is one of the most extensively used graph visualization platforms for retrieving and predicting PPIs, with information obtained from experimental data, bioinformatics prediction, literature mining, and multiple databases [30]. To achieve a comprehensive understanding of the interactions among proteins, the relevant targets were inputted into STRING (https://string-db.org/; Version 11.0) for PPI network construction. Based on the intrinsic scoring mechanism, the higher the score, the more reliable the predictions of the interactions among proteins. We set the minimum value for the confidence at 0.4 and removed the disconnected proteins from the network. After importing the interaction data downloaded from STRING into the Cytoscape visualization software, the final PPI network was obtained and the number of edges per protein was tallied. In the network, nodes refer to proteins, whereas edges represent interactions among proteins.

### GO and pathway enrichment analyses

The GO analysis can provide a standardized description and annotation for genes and gene products. Through GO analysis, users can obtain a comprehensive understanding from several aspects, including biological processes, cellular components, and molecular functions [31]. The Kyoto Encyclopedia of Genes and Genomes (KEGG) is a commonly used resource for the systematic analysis of gene functions and related high-level genome functional information [32, 33]. In the present study, GO and KEGG pathway enrichment analyses were conducted to explore the biological effects of *S. nigrum* using R the software (version 3.6) and three installed packages: (i) “DOSE,” an R package for semantic similarity computations among disease ontology terms and genes, allowing biologists to explore the similarities of gene functions from the perspective of disease [34]; (ii) “clusterProfiler,” an R package for comparing biological themes and enrichment analysis among gene clusters [35]; and (iii) “pathview,” an R package for data visualization and integration based on the known pathways [36]. The corresponding *p-value* of each enriched term or pathway was calculated and corrected with Benjaminiand-Hochberg method to produce False Discovery Rate (FDR).

### Construction of the *S. nigrum* pathway

To obtain an intuitive understanding of the mechanisms underlying the effect on *S. nigrum* in BC, an integrated “*S. nigrum* pathway” was manually developed based on the KEGG route map. Pathways not directly related to the disease were eliminated.

## Results

### Identification of active ingredients

In total, 39 ingredients of *S. nigrum* were retrieved through the TCMSP database query (Table A1). After identifying the ingredients that met the criteria of predicted OB ≥ 30% and DL ≥ 0.18, seven active ingredients were finally retrieved: diosgenin (MOL000546), medioresinol (MOL002058), solanocapsine (MOL007356), quercetin (MOL000098), cholesterol (MOL000953), beta-carotene (MOL002773), and sitosterol (MOL000359) (as shown in Table 1). These active ingredients were considered to be mainly responsible for the therapeutic effects of *S. nigrum*.

**Table 1:** The corresponding information and structures of the seven active components of *Solanum nigrum* with the predicted oral bioavailability (OB) ≥ 30% and drug likeness (DL) ≥ 0.18

No.	Molecule ID	Molecule name	MW	OB	DL	Parts	Structure
1	MOL000546	Diosgenin	414.69	80.88	0.81	leaves stems	
2	MOL002058	Medioresinol	388.45	57.2	0.62	fruits	
3	MOL007356	Solanocapsine	430.75	52.94	0.67	fruits	
4	MOL000098	Quercetin	302.35	46.43	0.28	leaves stems	
5	MOL000953	Cholesterol	386.73	37.87	0.68	fruits	
6	MOL002773	beta-Carotene	536.96	37.18	0.58	fruits	
7	MOL000359	Sitosterol	414.79	36.91	0.75	fruits	

### Target identification and analysis

After target fishing using the TCMSP database, 84 potential targets were predicted to be regulated by the seven active ingredients of *S. nigrum*. Subsequently, by searching the GeneCards and OMIM databases for genes related to the occurrence and development of BC, 7624 genes were obtained. Integration of the targets regulated by *S. nigrum* and the targets related to BC revealed that 77 genes overlapped (shown in Table A2). Therefore, it is reasonable for us to consider that these genes are the potential therapeutic targets of *S. nigrum* in BC.

### I–T network construction and analysis

**Fig. 2:**
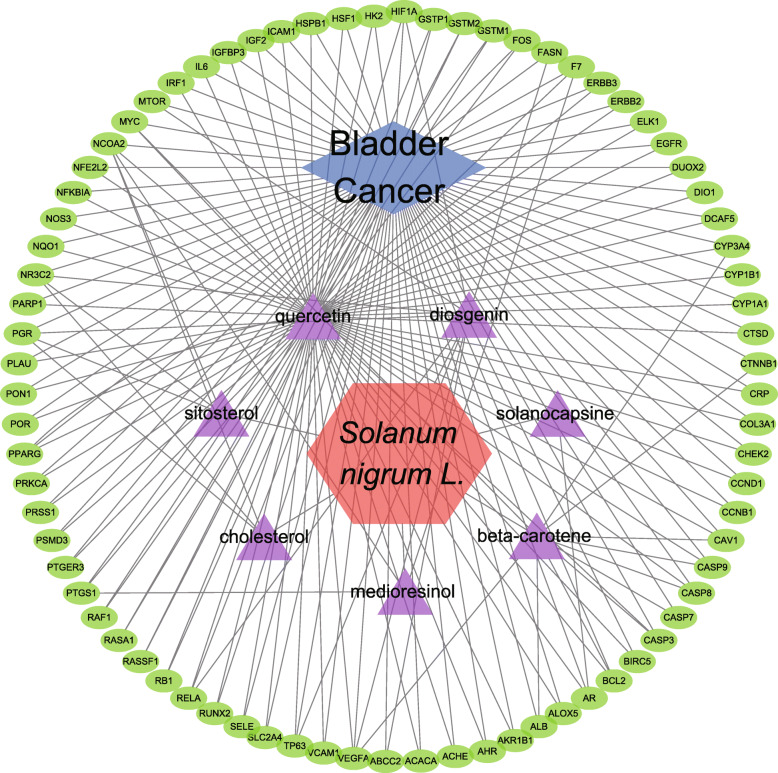
The ingredient-target network generated in this study. The green nodes represent potential targets related to BC, and the purple nodes represent the active herbal ingredients; lines represent the interactions between them

To better understand the interaction between the candidate ingredients and the potential targets of *S. nigrum* in BC, an I–T interaction network was constructed, in which each ingredient was linked to one or more target genes if the gene was a potential target of this ingredient. As shown in Fig. 2, the seven active ingredients were mapped to 77 potential target genes with 100 edges. The purple nodes represent the active ingredients and the green nodes represent the target genes. The edges indicate the interactions among them. Generally, the more genes an ingredient links to, the more likely it is to play a major role in the pharmacology of the herb. The degree of a node represents the number of routes connected to that node. The results of I–T network analysis showed that quercetin was connected to all targets with the highest degree value of 77, which suggested that quercetin was more central than other active herbal ingredients, followed by beta-carotene (11 degrees) and diosgenin (9 degrees). In addition, 14 genes (myc proto-oncogene [*MYC*], *HIF1α, F7, CYB3A4, CAV1, RELA,* caspase 3 [CAS*P3*]*, CASP8, CASP9, BCL2,* androgen receptor [*AR*]*, TP63, PTGS1*, and peroxisome proliferator activated receptor gamma [*PPARG*]) had two degrees, three genes (progesterone receptor [*PGR*], nuclear receptor subfamily 3 group C member 2 [*NR3C2*], and vascular endothelial growth factor A [*VEGFA*]) had three degrees, and one gene (nuclear receptor coactivator 2 [*NCOA2*]) had four degrees, indicating that these genes were targeted by more than one active herbal ingredient.

### PPI network construction and analysis

**Fig. 3:**
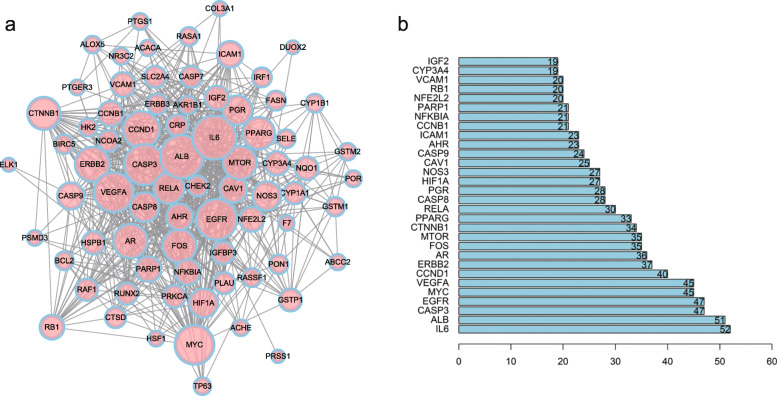
**a** The protein–protein interaction (PPI) network of the potential targets associated with *Solanum nigrum* related to bladder cancer (BC). The nodes represent proteins, and the edges represent the interactions among them. The larger the node, the greater the degree of the node. **b** The bar plot of the first 30 target proteins sorted based on degree value. The longer the bar, the greater the connection of the protein

A PPI network of the targets of *S. nigrum* in the treatment of BC was constructed to analyze the interactions among proteins and to mine hub proteins. After setting the confidence level to a value equal to or higher than 0.4 and after eliminating any protein not connected to the network, we obtained 77 proteins with 697 interrelations in the PPI network (Fig. 3a). A larger node size indicates a gene with a greater degree, which reflects a prioritization of the protein. Additionally, the gene with a prior degree value has been ranked in Fig. 3b. The degree value of interleukin 6 (IL-6) was 52, which was significantly higher than those of other proteins, indicating that IL-6 could act as a hub in the network. Besides, many proteins involved in tumor development in BC also showed high degree values, such as CASP3 and epidermal growth factor receptor (EGFR) (degrees of both are 47), MYC and VEGFA (degrees of both are 45), cyclin D1 (CCND1) (degree = 40), erb-b2 receptor tyrosine kinase 2 (ERBB2) (degree = 37), mechanistic target of rapamycin kinase (mTOR) (degree = 34), PPARG (degree = 33) and so on.

### GO functional enrichment analysis

**Fig. 4:**
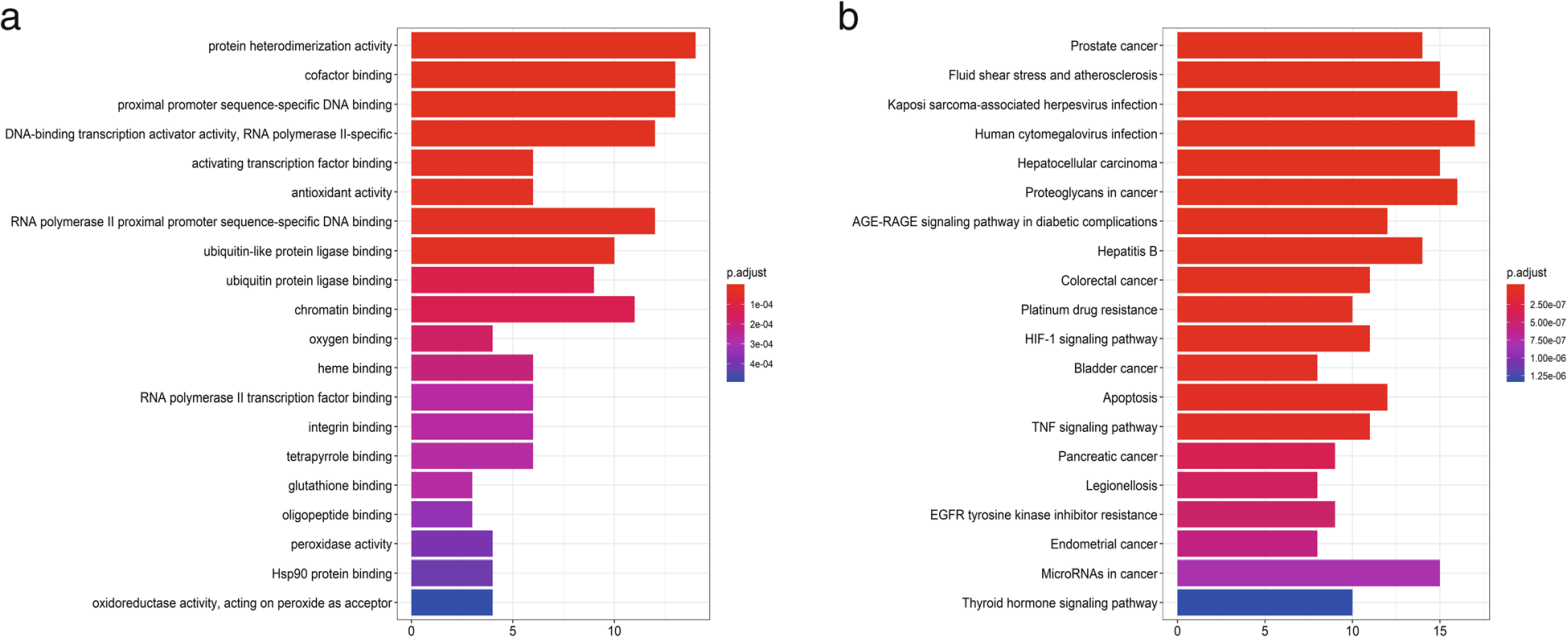
**a** Gene ontology (GO) analysis of the target genes associated with *Solanum nigrum* related to bladder cancer (BC); **b** KEGG pathway enrichment analysis of the target genes related to BC that are modulated by *S. nigrum*. The x-axis represents significant enrichment counts of these terms, and the y-axis represents the categories of GO terms and KEGG pathways of the target genes (*p*-value ≤0.01)

GO enrichment analysis was performed to elucidate the biological processes that the 77 genes participated in. Eighty-six remarkably enriched GO terms of these genes (*p*-value ≤0.01) were obtained. The 20 most significantly enriched GO terms are presented in Fig. 4a. The counts, *p*-values, and FDR of the significant terms are presented in Table A3. In summary, numerous genes were combined into one set, and many gene sets regulated specific biological activities involved in the occurrence and development of BC, such as protein heterodimerization activity (GO: 0046982), antioxidant activity (GO: 0016209), positive transcriptional regulation by RNA polymerase II promoter (GO: 0001085), nuclear receptor activity (GO: 0004879), and kinase regulator activity (GO: 0019207).

### KEGG pathway enrichment analysis

The pathways related to the activity of *S. nigrum* in BC treatment were elucidated by KEGG pathway enrichment analysis. In total, 89 pathways were primarily enriched. The 20 most significantly enriched pathways (*p*-value ≤0.01) are presented in Fig. 4b. The counts, *p*-values, and FDRs of the significant pathways are presented in Table A4. In summary, these genes were enriched in multiple pathways known to contribute to the tumorigenesis and progression of BC, such as the HIF-1 signaling pathway (hsa04066), bladder cancer pathway (hsa05219), apoptosis pathway (hsa04210), tumor necrosis factor (TNF) signaling pathway (hsa04668), p53 signaling pathway (hsa04115), mitogen-activated protein kinase (MAPK) signaling pathway (hsa04010), and PI3K/Akt signaling pathway (hsa04151).

### Integrated *S. nigrum* pathway construction

**Fig. 5:**
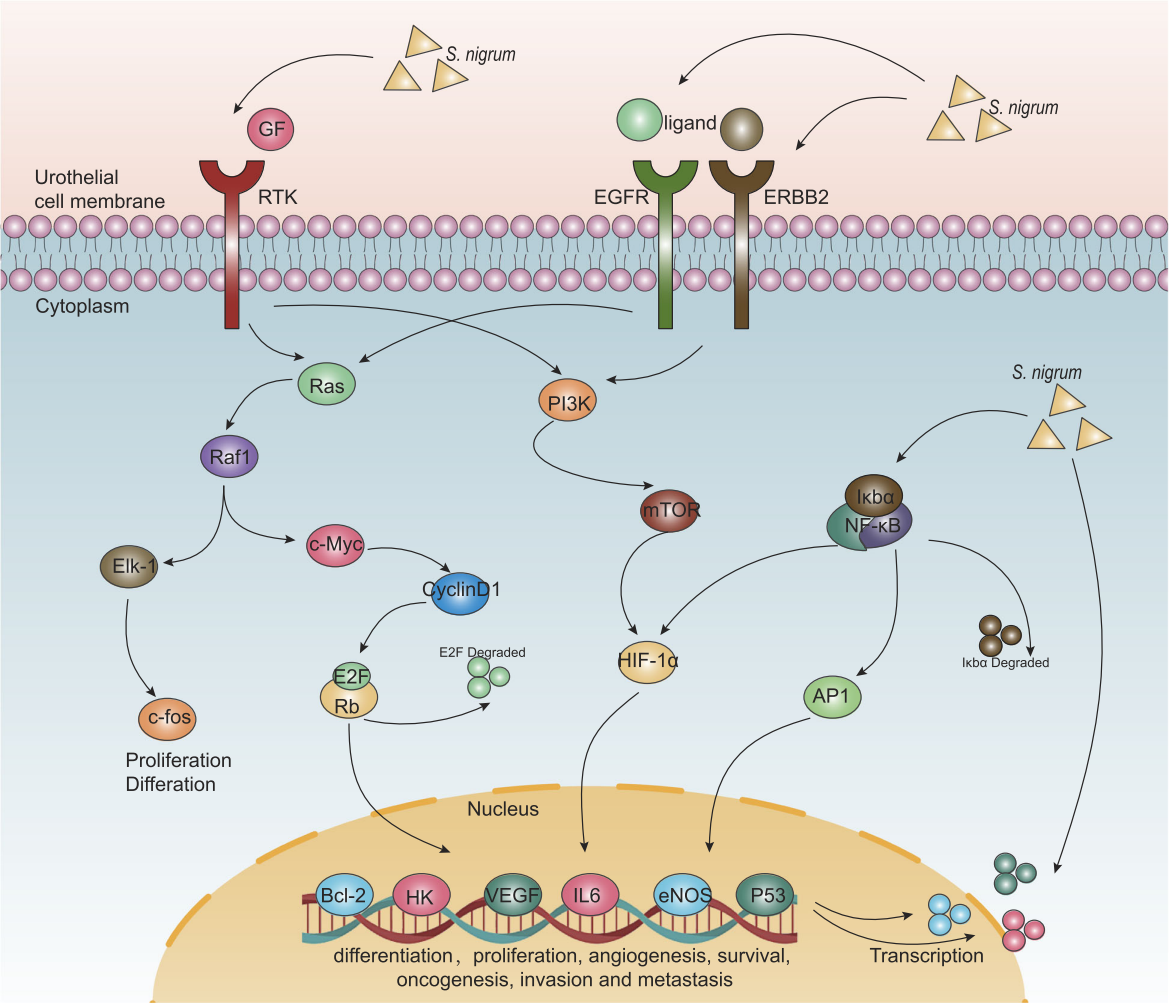
The *S. nigrum* pathway constructed in the present study. Abbreviations: GF, growth factor; RTK, receptor tyrosine kinases; EGF, epidermal growth factor; EGFR, epidermal growth factor receptor; ERBB2, Erb-B2 receptor tyrosine kinase 2; Ras, HRas & KRas proto-oncogene; Raf-1, threonine-protein kinase Raf1; Elk-1, ETS transcription factor ELK1; c-Myc, MYC Proto-Oncogene; c-fos, AP-1 transcription factor subunit; Rb, protein phosphatase 1; E2F, retinoblastoma-associated protein; PI3K, phosphoinositide 3-kinase; mTOR, mechanistic target of rapamycin kinase; HIF-1α hypoxia-inducible factor 1 subunit alpha; NF-κB, nuclear factor kappa B; IκBα, NF-κB inhibitor alpha; AP-1, Jun & FOS proto-oncogene; Bcl-2, Bcl-2 apoptosis regulator; HK, hexokinase; VEGF, vascular endothelial growth factor A; eNOS, endothelial nitric oxide synthase; IL-6, interleukin 6; P53, tumor protein P53

To further comprehensively reveal the molecular mechanisms underlying the effects of *S. nigrum* against BC, an integrated *S. nigrum* pathway model was assembled by combining the significant pathways associated with the occurrence and development of BC identified through KEGG pathway enrichment analysis and some targets identified by I–T network analysis. Detailed information about the representative therapeutic modules to clarify the mechanisms of action of *S. nigrum* in BC was presented in Fig. 5. *S. nigrum* may exert anti-tumor effects in BC patients mainly by regulating the BC pathway (hsa05219), MAPK signaling pathway (hsa04010), HIF-1 signaling pathway (hsa04066), TNF signaling pathway (hsa04668), apoptosis signaling pathway (hsa04210), PI3K-Akt signaling pathway (hsa04151), p53 signaling pathway (hsa04115), and NF-kappa B signaling pathway (hsa04064).

## Discussion

BC is a heterogeneous disease [3] and the fifth most common malignancy globally [2]. Considering the characteristics of BC, a key challenge in its treatment is the prevention of recurrence and progression to advanced stages [2, 8]. Although significant advances have been made in BC therapy with the advent of targeted therapies and immunotherapy, only a minority of patients benefit from the treatment and patient outcomes remain poor [3]. As a valuable alternative medicine, TCM has been associated with rich theoretical resources and invaluable experience that can be applied in clinical cancer therapy, and it has served as a vital resource for the development of multi-target drugs [19]. *S. nigrum* has been used to treat BC effectively [11]. Considering the component diversity of this herb, we used network pharmacology to comprehensively explore the mechanism of action of *S. nigrum* in BC treatment.

Via systems pharmacologic analysis, seven active ingredients were finally selected and 77 common target genes for BC were predicted. The seven identified bioactive compounds, including quercetin, sitosterol, diosgenin, medioresinol, beta-carotene, cholesterol and solanocapsine, are the major pharmacological constituents of *S. nigrum*. Quercetin, a flavonoid, belongs to an extensive class of polyphenolic compounds. It can not only inhibit the apoptosis and proliferation of numerous malignant cells, including BC and tumor growth in animal models through multiple signaling pathways [37], such as the AMPK [38], PI3K/Akt [39], and JNK pathways [40], but can also enhance the sensitivity of other anti-cancer agents and reverse the drug resistance of cancer cells [40]. Based on the I–T network, quercetin was linked to all targets with the highest degree among all selected ingredients, suggesting potential synergies between quercetin and all the other ingredients. Quercetin may act here as the main synergistic ingredient to increase the efficacy of *S. nigrum* as an anti-tumor agent. In addition, quercetin exhibits pharmacological activities as radical-scavenging antioxidant activity and anti-inflammatory activity to inhibit carcinogenesis [41] . In clinical practice, a combination of cyclophosphamide and quercetin has been demonstrated to have good antineoplastic and antifatigue effects, as well as an excellent safety profile, in patients with BC [42].

Numerous studies have demonstrated that sitosterol exhibits potent anti-cancer activity against various malignancies, such as lung, breast, gastric, colon, and prostate cancers. Its potential mechanisms of action inducing apoptosis and inhibiting epithelial–mesenchymal transition through the blockade of multiple signaling pathways, including the MAPK, PI3K/Akt, Bcl-2, and Akt/GSK-3β signaling pathways [43]. Diosgenin is a typical traditional medicine with multiple bioactivities, such as antidiabetic, anti-inflammatory, and anti-tumor properties [44]. Reportedly, most steroidal medicines are semisynthetic products obtained predominantly from diosgenin [45]. Medioresinol is a member of the lignan family, which are the predominant sources of phytoestrogens in western diets [46].

Dietary intake of plant lignans is beneficial for treating esophageal adenocarcinoma [47], colorectal cancer [48], and cardiovascular diseases and in the risk of hormone-dependent cancers, such as breast, prostate, and endometrial cancers [46]. Beta-carotene mediates its therapeutic effects on human neuroblastoma, including the inhibition of cancer stemness and metastasis, by suppressing the enzyme activity and expression of MMPs, as well as the expression of HIF-1α and its downstream targets [49]. Notably, unlike the other active ingredients, cholesterol in high content promotes inflammatory environments via the production of different cytokines, such as CXCL9 and CXCL10, but it also characterizes tumors, including breast, prostate, ovarian, lymphoma, colorectal cancer, and renal cell carcinoma, with poor prognosis [50]. Solanocapsine, a steroidal alkaloid, has shown potential therapeutic value as an inhibitor of acetylcholinesterase in patients with Alzheimer’s disease [51]; however, its anti-cancer pharmacological effects are yet to be studied.

According to the I–T network, we observed that many genes were linked with at least two ingredients, indicating the synergistic properties of different ingredients of *S. nigrum* on BC. In our study, *NCOA2*, also commonly referred to as transcriptional intermediate factor 2, was predicted to be targeted by four active ingredients, including quercetin, sitosterol, medioresinol, and cholesterol. The activation of *NCOA2* contributes to the progression of BC, which is closely related to the activation of the EGFR and AR pathways [41]. In addition, the knockdown of *NCOA2* results in a decrease in androgen-mediated cell proliferation in BC cell lines [42]. Preclinical evidence also reveals that AR inhibition can inhibit cancer cell growth and improve outcomes in patients with BC [3]. Therefore, *NCOA2* is an attractive therapeutic target in BC treatment, which is largely consistent with our results. Moreover, three genes, *VEGF*, *PGR*, and *NR3C2*, are linked to three active ingredients, respectively. *VEGF* is a well-known therapeutic target for many malignancies, including BC [52, 53]. Increased *PGR* mRNA expression has been evaluated for its predictive value for non-muscle-invasive [54] and muscle-invasive BC [55], and this well-established BC marker is also a potential therapeutic target for BC [56]. *NR3C2* has been described as a tumor suppressor in multiple malignancies, such as pancreatic, lung, colon, and renal cancers [57]; however, the prognostic value and biological effect of low *NR3C2* level in BC remains unclear [58].

PPI network analysis revealed that the degree value of IL-6 was the highest, implying its role as a hub. The pro-inflammatory cytokine IL-6 has been established as one of the most biologically active cytokines with multiple biological functions [19]. Numerous studies have demonstrated that the aberrant expression of IL-6 and its receptor correlates with malignant phenotypes of multiple tumors, as well as the diagnosis, prognosis, and treatment of cancer [59]. In one study, IL-6 was overexpressed in BC tissues compared with that in non-malignant tissues, at both mRNA and protein levels, and was significantly associated with advanced clinical stage, high recurrence, and poor survival rate, indicating the diagnostic significance of IL-6 in BC [60]. Meanwhile, targeting IL-6 might be a promising therapeutic target in BC because IL-6 inhibition can attenuate bladder tumor growth and invasive capability [60]. *S. nigrum* can also reduce inflammation [61]. Therefore, targeting IL-6 is potentially one of the most effective pathways of *S. nigrum*-induced therapeutic effects on BC.

In addition to IL-6, many proteins involved in the occurrence and development of BC also showed high degree values, such as CASP3, EGFR, MYC, VEGFA, CCND1, ERBB2, mTOR and PPARG. As known, CASP3 is a key enzyme in the execution of apoptosis and CCND1 codes for the cyclin D1 protein to affect the cell cycle progression [62], both of which were reported to contribute to many types of cancers and closely associated with cell motility, invasion, and metastasis [63]. EGFR overexpression is also found in a variety of human malignancies [64], including BC, which is implicated in stimulation of BC cell growth and induction of apoptosis [65] and appears to be a feasible target for BC therapy. ERBB2 also belongs to the EGFR family, which overexpression is associated with vascular growth and features of biological aggressiveness of BC [66]. MYC is known to function as an oncogene involved in the activation of various oncogenic signaling pathways, the inhibition of which can suppress cell growth and migration in BC [67] and cisplatin resistant BC cells [68]. PPARG could promote differentiation and regulate expression of mitochondrial genes in bladder epithelial cells, and its activation contributes to bladder carcinogenesis [69]. The importance of mTOR in carcinogenesis is becoming evident. In addition to relaying the oncogenic signals from the upstream PI3K/AKT pathway in various cancers, mTOR may play a direct role in human tumorigenesis if mutated, including in BC, which further support the view of mTOR as one of the major therapeutic target against cancer [70]. These evidences all provide insights into the mechanistic function of *S. nigrum* as a bladder tumor suppressor.

Based on the GO classification and KEGG pathway enrichment results, *S. nigrum* interfered with the malignant phenotype of BC through diverse biological processes and signaling pathways. For example, the GO terms “RNA polymerase II,” “protein heterodimerization activity,” and “antioxidant activity” were closely associated with bladder carcinogenesis [71–73]. Among the enriched pathways, NF-κB signaling pathway (hsa04064, degree = 7) was confirmed to contribute to tumorigenicity and correlate with progression and prognosis in BC [74] and to participate in the metastasis-inhibiting effects of *S. nigrum* on melanoma cells [16]. The MAPK signaling pathway (hsa04010, degree = 14), as one of the most intensively studied signaling pathways, has been found to be deregulated in various diseases, ranging from inflammatory and immunological syndromes to cancer, and controls the growth, proliferation, differentiation, and survival of various cells [75]. The results of an in vivo experiment suggest that targeting the MAPK pathway could be an effective therapy for BC [76]. In addition, *S. nigrum* could attenuate the malignant phenotype and tumor growth through the MAPK/mTOR signaling pathway in hepatocellular carcinoma [13] and the p38 signaling pathway in prostate cancer cells [77]. Therefore, MAPK signaling may be a crucial therapeutic target in the anti-tumor effects of *S. nigrum*. Furthermore, the upregulation of EGFR and ERBB2 protein levels was detected in numerous muscle-invasive BC samples, which induced PI3K (hsa04151, degree = 15) and Ras activation, and were associated with BC grade, stage, and outcome [76]. As predicted in our study, EGFR and ERBB2 have been suggested to be targeted by quercetin [73]. Moreover, *S. nigrum* decreased blood serum TNF-α levels, which is consistent with the triggering of apoptosis in tumor cells [78]. Angiogenesis also plays a major role in different stages of tumor progression; therefore, some related pathways, such as the VEGF signaling pathway (hsa04370, degree = 6) and the HIF-1 signaling pathway (hsa04066, degree = 11), have been identified as the target pathways for treating BC [53, 79]. All the results above are highly consistent with our findings from the GO and KEGG analyses. By integrating several previous well-established BC-related pathways and the target genes identified by I–T network analysis, we assembled a proposed *S. nigrum* pathway model (Fig. 5). In theory *S. nigrum* might exert anti-BC effects mainly through acting on MAPK, HIF-1, TNF, PI3K-Ak, P53, NF-kappa B and apoptosis signaling pathways, which are importantly implicated in tumor cell growth, angiogenesis, invasion and metastasis, by directly regulating the expression of *EGFR, EERB2, GF, RAS, RAF1, ELK1, FOS, MYC, CCND1, mTOR, HIF1α, NFκB, BCL2, HK, VEGF* and *IL-6.* We hope that the pathway model facilitates a better understanding of the pharmacological mechanisms underlying the anti-cancer effects mediated by *S. nigrum* based on a comprehensive perspective.

*S. nigrum* has been reported to have dose-dependent toxic effects, resulting in nausea, vomiting, diarrhea, dizziness, headache, fever, sweating, tachycardia, loss of speech, blindness, mental confusion, coma, and death [80, 81]. A recent study reported that a patient suffered acute interstitial nephritis following the ingestion of *S. nigrum* and was successfully treated using corticosteroid therapy [81]. *S. nigrum* toxicity is mainly attributed to the presence of glycoalkaloids, including solanine, solasonine, and solamargine, which constitute the plant’s primary natural defenses, as these compounds are toxic even at relatively low quantities [80]. *S. nigrum* has also been used to treat pain, inflammation, and fever [80]. Considering its widespread application for disparate ailments, more studies should be conducted to assess its toxicity and appropriate therapeutic dosage, as well as to explore standardized preparation methods, which would facilitate the optimization of therapeutic strategies, minimize side effects, and extend its potential benefits to more patients.

The present study had some limitations. First, the bioactive ingredients identified could differ from those actually absorbed by patients with BC. Secondly, it is difficult to distinguish the inhibitory target genes from the activated genes. In addition, not all predictions in the present study have been verified experimentally. Therefore, experimental verification of the active herbal ingredients is required to validate the hypotheses further.

## Conclusions

The network pharmacology approach provides a predicative novel strategy for seeking evidence for the action mechanisms of herbal medicines based on a holistic perspective. Following target fishing and I–T network analysis, seven active ingredients acting on 77 BC-related genes were predicted, suggesting that *S. nigrum* might exert pharmacological effects against BC through multi-targets. Quercetin linked to the large number of target genes may act as the main synergistic ingredient in *S. nigrum*, and *NCOA2* was uniquely targeted by four ingredients. In addition, PPI network analysis indicated the hub role of IL-6. Additionally, gene enrichment analysis demonstrated that the active ingredients have potential therapeutic benefits with regard to BC via multiple pathways, such as the HIF-1, TNF, P53, MAPK, PI3K/Akt, apoptosis, and bladder cancer pathways. Nevertheless, further experiments are required to validate the theoretical predictions.

## Supplementary Information

**Additional file 1: Table A1.** Thirty-nine components of *Solanum nigrum* and their corresponding molecular weight (MW), predicted oral bioavailability (OB) and drug likeness (DL) scores. **Table A2.** Bladder cancer (BC) related targets in *Solanum nigrum*. By combining the related targets of active ingredients screened from *S. nigrum* and the disease-related targets, 100 overlapping targets (involving 23 duplicates) were selected as the key targets involved in the treatment of BC. **Table A3.** Gene ontology (GO) terms of therapeutic target genes and their corresponding counts, *p-*value, and FDR. Through the GO enrichment of the key targets, 86 remarkably enriched (*p-*value ≤0.01) GO terms were obtained, indicating that some targets were involved in tumorigenesis. **Table A4.** Kyoto Encyclopedia of Genes and Genomes (KEGG) pathway of therapeutic target genes and their corresponding counts, *p*-value, and FDR. Through KEGG enrichment of the key targets, 89 remarkably enriched (*p-*value ≤0.01) pathways were obtained, indicating that numerous targets are involved in the processes of tumorigenesis and tumor progression.

## Data Availability

The datasets used and analyzed during the current study are available from the corresponding author on reasonable request.

## Abbreviations

BC: Bladder urothelial carcinoma
TCM: Traditional Chinese medicine
OB: Oral bioavailability
DL: Drug likeness
GO: Gene Ontology
KEGG: Kyoto Encyclopedia of Genes and Genomes
OMIM: Online Mendelian Inheritance in Man
FDR: False Discovery Rate
*MYC*: Myc proto-oncogene
*CASP3*: Caspase 3
*AR*: Androgen receptor
*PGR*: Progesterone receptor
*PPARG*: Peroxisome proliferator activated receptor gamma
*NR3C2*: Nuclear receptor subfamily 3 group C member 2
*VEGFA*: Vascular endothelial growth factor A
*NCOA2*: Nuclear receptor coactivator 2
IL-6: Interleukin 6
EGFR: Epidermal growth factor receptor
CCND1: Cyclin D1
ERBB2: Erb-b2 receptor tyrosine kinase 2
mTOR: Mechanistic target of rapamycin kinase
