# Supplementary material for: A network pharmacology perspective for deciphering potential mechanisms of action of Solanum nigrum L. in bladder cancer
Source: BMC Complement Med Ther. 2021 Jan 25;21:45. doi: 10.1186/s12906-021-03215-3 (PMC7836472; doi:10.1186/s12906-021-03215-3)
Supplement: Supplementary file 1 — Additional file 1: Table A1. Thirty-nine components of Solanum nigrum and their corresponding molecular weight (MW), predicted oral bioavailability (OB) and drug likeness (DL) scores. Table A2. Bladder cancer (BC) related targets in Solanum nigrum. By combining the related targets of active ingredients screened from S. nigrum and the disease-related targets, 100 overlapping targets (involving 23 duplicates) were selected as the key targets involved in the treatment of BC. Table A3. Gene ontology (GO) terms of therapeutic target genes and their corresponding counts, p-value, and FDR. Through the GO enrichment of the key targets, 86 remarkably enriched (p-value ≤0.01) GO terms were obtained, indicating that some targets were involved in tumorigenesis. Table A4. Kyoto Encyclopedia of Genes and Genomes (KEGG) pathway of therapeutic target genes and their corresponding counts, p-value, and FDR. Through KEGG enrichment of the key targets, 89 remarkably enriched (p-value ≤0.01) pathways were obtained, indicating that numerous targets are involved in the processes of tumorigenesis and tumor progression. [file 12906_2021_3215_MOESM1_ESM.docx]

**Appendices**

**Table A1** Thirty-nine components of *Solanum nigrum* and their corresponding molecular weight (MW), predicted oral bioavailability (OB) and drug likeness (DL) values.

| **No.** | **Mol ID** | **Molecule name** | **MW** | **OB** | **DL** |
| --- | --- | --- | --- | --- | --- |
| 1 | MOL000103 | 4-Hydroxybenzoic acid | 138.13 | 30.15 | 0.03 |
| 2 | MOL000105 | [Protocatechuic acid](http://lsp.nwu.edu.cn/molecule.php?qn=105) | 154.13 | 25.37 | 0.04 |
| 3 | MOL000114 | [Vanillic acid](http://lsp.nwu.edu.cn/molecule.php?qn=114) | 168.16 | 35.47 | 0.04 |
| 4 | MOL001787 | Adenosine | 267.28 | 15.98 | 0.18 |
| 5 | MOL001842 | [Pinoresinol](http://lsp.nwu.edu.cn/molecule.php?qn=1842) | 358.42 | 4.25 | 0.52 |
| 6 | MOL002058 | Medioresinol | 388.45 | 57.2 | 0.62 |
| 7 | MOL002773 | [beta-Carotene](http://lsp.nwu.edu.cn/molecule.php?qn=2773) | 536.96 | 37.18 | 0.58 |
| 8 | MOL000359 | [Sitosterol](http://lsp.nwu.edu.cn/molecule.php?qn=359) | 414.79 | 36.91 | 0.75 |
| 9 | MOL000396 | [(+)-Syringaresinol](http://lsp.nwu.edu.cn/molecule.php?qn=396) | 418.48 | 3.29 | 0.72 |
| 10 | MOL000040 | [Scopoletol](http://lsp.nwu.edu.cn/molecule.php?qn=40) | 192.18 | 27.77 | 0.08 |
| 11 | MOL004368 | [Hyperin](http://lsp.nwu.edu.cn/molecule.php?qn=4368) | 464.41 | 6.94 | 0.77 |
| 12 | MOL000437 | [Hirsutrin](http://lsp.nwu.edu.cn/molecule.php?qn=437) | 464.41 | 1.86 | 0.77 |
| 13 | MOL004448 | [Solatubin](http://lsp.nwu.edu.cn/molecule.php?qn=4448) | 397.71 | 17.12 | 0.76 |
| 14 | MOL004495 | [Tigogenin](http://lsp.nwu.edu.cn/molecule.php?qn=4495) | 416.71 | 14.09 | 0.81 |
| 15 | MOL000546 | [Diosgenin](http://lsp.nwu.edu.cn/molecule.php?qn=546) | 414.69 | 80.88 | 0.81 |
| 16 | MOL006347 | [(2R)-6-(2-Hydroxyethyl)-2,5,7-trimethyl-1-indanone](http://lsp.nwu.edu.cn/molecule.php?qn=6347) | 218.32 | 46.6 | 0.09 |
| 17 | MOL000663 | [Lignoceric acid](http://lsp.nwu.edu.cn/molecule.php?qn=663) | 368.72 | 14.9 | 0.33 |
| 18 | MOL006850 | [C10822](http://lsp.nwu.edu.cn/molecule.php?qn=6850) | 413.71 | 14.63 | 0.81 |
| 19 | MOL006851 | [Solamargine](http://lsp.nwu.edu.cn/molecule.php?qn=6851) | 577.89 | 10.9 | 0.29 |
| 20 | MOL006852 | [Solamargine_qt](http://lsp.nwu.edu.cn/molecule.php?qn=6852) | 415.73 | 16.05 | 0.8 |
| 21 | MOL006853 | [Solasonine](http://lsp.nwu.edu.cn/molecule.php?qn=6853) | 884.19 | 25.94 | 0.06 |
| 22 | MOL006854 | [Solasonine_qt](http://lsp.nwu.edu.cn/molecule.php?qn=6854) | 413.71 | 16.88 | 0.81 |
| 23 | MOL006857 | [Tomatidenol](http://lsp.nwu.edu.cn/molecule.php?qn=6857) | 413.71 | 10.68 | 0.81 |
| 24 | MOL007158 | [Spirostan-3-ol, (3beta,5alpha,25S)-](http://lsp.nwu.edu.cn/molecule.php?qn=7158) | 416.71 | 13.16 | 0.81 |
| 25 | MOL007351 | [12β,27-dihydroxysolasodine](http://lsp.nwu.edu.cn/molecule.php?qn=7351) | 445.71 | 11.72 | 0.76 |
| 26 | MOL007352 | [23-o-Acetyl-12β-hydroxysolasodine](http://lsp.nwu.edu.cn/molecule.php?qn=7352) | 487.75 | 12.17 | 0.63 |
| 27 | MOL007353 | [Solamargine](http://lsp.nwu.edu.cn/molecule.php?qn=7353) | 868.19 | 30.01 | 0.04 |
| 28 | MOL007354 | [Uttronin A](http://lsp.nwu.edu.cn/molecule.php?qn=7354) | 1035.32 | 11.91 | 0.02 |
| 29 | MOL007355 | [Acetylcholine](http://lsp.nwu.edu.cn/molecule.php?qn=7355) | 146.24 | 27.8 | 0.02 |
| 30 | MOL007356 | [Solanocapsine](http://lsp.nwu.edu.cn/molecule.php?qn=7356) | 430.75 | 52.94 | 0.67 |
| 31 | MOL007357 | [Ac-Shanz-ME](http://lsp.nwu.edu.cn/molecule.php?qn=7357) | 448.47 | 15.84 | 0.59 |
| 32 | MOL007358 | [Uttroside a_qt](http://lsp.nwu.edu.cn/molecule.php?qn=7358) | 286.31 | 11.51 | 0.17 |
| 33 | MOL007359 | [Pterosin B glucoside](http://lsp.nwu.edu.cn/molecule.php?qn=7359) | 380.48 | 16.07 | 0.46 |
| 34 | MOL007360 | [Quercetin-3-gentiobioside](http://lsp.nwu.edu.cn/molecule.php?qn=7360) | 626.57 | 3.45 | 0.64 |
| 35 | MOL007361 | [Desgalactotigonin](http://lsp.nwu.edu.cn/molecule.php?qn=7361) | 1035.32 | 11.5 | 0.02 |
| 36 | MOL007362 | [Spirostan-3-ol, (3alpha,5alpha,25S)-](http://lsp.nwu.edu.cn/molecule.php?qn=7362) | 416.71 | 16.88 | 0.81 |
| 37 | MOL007363 | [Solanaviol](http://lsp.nwu.edu.cn/molecule.php?qn=7363) | 382.45 | 15.63 | 0.44 |
| 38 | MOL000953 | [Cholesterol](http://lsp.nwu.edu.cn/molecule.php?qn=953) | 386.73 | 37.87 | 0.68 |
| 39 | MOL000098 | [Quercetin](http://lsp.nwu.edu.cn/molecule.php?qn=98) | 302.25 | 46.43 | 0.28 |

**Table A2** Bladder cancer (BC) related targets in *Solanum nigrum*. By combining the related targets of active ingredients screened from *S. nigrum* and the disease-related targets, 100 overlapping targets (involving 23 duplicates) were selected as the key targets involved in the treatment of BC.

| No. | MolId | MolName | Target | Symbol |
| --- | --- | --- | --- | --- |
| 1 | MOL000546 | Diosgenin | Progesterone receptor | *PGR* |
| 2 | MOL000546 | Diosgenin | Mineralocorticoid receptor | *NR3C2* |
| 3 | MOL000546 | Diosgenin | Transcription factor p65 | *RELA* |
| 4 | MOL000546 | Diosgenin | Vascular endothelial growth factor A | *VEGFA* |
| 5 | MOL000546 | Diosgenin | Cellular tumor antigen p53 | *TP63* |
| 6 | MOL000546 | Diosgenin | Fatty acid synthase | *FASN* |
| 7 | MOL000546 | Diosgenin | Hypoxia-inducible factor 1-alpha | *HIF1A* |
| 8 | MOL000546 | Diosgenin | Canalicular multi-specific organic anion transporter 1 | *ABCC2* |
| 9 | MOL000546 | Diosgenin | Serine/threonine-protein kinase mTOR | *MTOR* |
| 10 | MOL002058 | Medioresinol | Prostaglandin G/H synthase 1 | *PTGS1* |
| 11 | MOL002058 | Medioresinol | Nuclear receptor coactivator 2 | *NCOA2* |
| 12 | MOL002058 | Medioresinol | Coagulation factor VII | *F7* |
| 13 | MOL007356 | Solanocapsine | Androgen receptor | *AR* |
| 14 | MOL000098 | Quercetin | Prostaglandin G/H synthase 1 | *PTGS1* |
| 15 | MOL000098 | Quercetin | Androgen receptor | *AR* |
| 16 | MOL000098 | Quercetin | Peroxisome proliferator activated receptor gamma | *PPARG* |
| 17 | MOL000098 | Quercetin | Nuclear receptor coactivator 2 | *NCOA2* |
| 18 | MOL000098 | Quercetin | Aldose reductase | *AKR1B1* |
| 19 | MOL000098 | Quercetin | Trypsin-1 | *PRSS1* |
| 20 | MOL000098 | Quercetin | Coagulation factor VII | *F7* |
| 21 | MOL000098 | Quercetin | Acetylcholinesterase | *ACHE* |
| 22 | MOL000098 | Quercetin | Transcription factor p65 | *RELA* |
| 23 | MOL000098 | Quercetin | Epidermal growth factor receptor | *EGFR* |
| 24 | MOL000098 | Quercetin | Vascular endothelial growth factor A | *VEGFA* |
| 25 | MOL000098 | Quercetin | G1/S-specific cyclin-D1 | *CCND1* |
| 26 | MOL000098 | Quercetin | Apoptosis regulator Bcl-2 | *BCL2* |
| 27 | MOL000098 | Quercetin | Proto-oncogene c-Fos | *FOS* |
| 28 | MOL000098 | Quercetin | Caspase-9 | *CASP9* |
| 29 | MOL000098 | Quercetin | Urokinase-type plasminogen activator | *PLAU* |
| 30 | MOL000098 | Quercetin | Retinoblastoma-associated protein | *RB1* |
| 31 | MOL000098 | Quercetin | Interleukin-6 | *IL6* |
| 32 | MOL000098 | Quercetin | Caspase-3 | *CASP3* |
| 33 | MOL000098 | Quercetin | Cellular tumor antigen p53 | *TP63* |
| 34 | MOL000098 | Quercetin | ETS domain-containing protein Elk-1 | *ELK1* |
| 35 | MOL000098 | Quercetin | NF-kappa-B inhibitor alpha | *NFKBIA* |
| 36 | MOL000098 | Quercetin | NADPH-cytochrome P450 reductase | *POR* |
| 37 | MOL000098 | Quercetin | Caspase-8 | *CASP8* |
| 38 | MOL000098 | Quercetin | RAF proto-oncogene serine/threonine-protein kinase | *RAF1* |
| 39 | MOL000098 | Quercetin | Protein kinase C alpha type | *PRKCA* |
| 40 | MOL000098 | Quercetin | Hypoxia-inducible factor 1-alpha | *HIF1A* |
| 41 | MOL000098 | Quercetin | Receptor tyrosine-protein kinase erbB-2 | *ERBB2* |
| 42 | MOL000098 | Quercetin | Peroxisome proliferator-activated receptor gamma | *PPARG* |
| 43 | MOL000098 | Quercetin | Acetyl-CoA carboxylase 1 | *ACACA* |
| 44 | MOL000098 | Quercetin | Cytochrome P450 3A4 | *CYP3A4* |
| 45 | MOL000098 | Quercetin | Caveolin-1 | *CAV1* |
| 46 | MOL000098 | Quercetin | Myc proto-oncogene protein | *MYC* |
| 47 | MOL000098 | Quercetin | Cytochrome P450 1A1 | *CYP1A1* |
| 48 | MOL000098 | Quercetin | Intercellular adhesion molecule 1 | *ICAM1* |
| 49 | MOL000098 | Quercetin | E-selectin | *SELE* |
| 50 | MOL000098 | Quercetin | Vascular cell adhesion protein 1 | *VCAM1* |
| 51 | MOL000098 | Quercetin | Prostaglandin E2 receptor EP3 subtype | *PTGER3* |
| 52 | MOL000098 | Quercetin | Baculoviral IAP repeat-containing protein 5 | *BIRC5* |
| 53 | MOL000098 | Quercetin | Dual oxidase 2 | *DUOX2* |
| 54 | MOL000098 | Quercetin | Nitric oxide synthase, endothelial | *NOS3* |
| 55 | MOL000098 | Quercetin | Heat shock protein beta-1 | *HSPB1* |
| 56 | MOL000098 | Quercetin | Cytochrome P450 1B1 | *CYP1B1* |
| 57 | MOL000098 | Quercetin | G2/mitotic-specific cyclin-B1 | *CCNB1* |
| 58 | MOL000098 | Quercetin | Arachidonate 5-lipoxygenase | *ALOX5* |
| 59 | MOL000098 | Quercetin | Glutathione S-transferase P | *GSTP1* |
| 60 | MOL000098 | Quercetin | Nuclear factor erythroid 2-related factor 2 | *NFE2L2* |
| 61 | MOL000098 | Quercetin | NAD(P)H dehydrogenase [quinone] 1 | *NQO1* |
| 62 | MOL000098 | Quercetin | Poly [ADP-ribose] polymerase 1 | *PARP1* |
| 63 | MOL000098 | Quercetin | Aryl hydrocarbon receptor | *AHR* |
| 64 | MOL000098 | Quercetin | 26S proteasome non-ATPase regulatory subunit 3 | *PSMD3* |
| 65 | MOL000098 | Quercetin | Solute carrier family 2, facilitated glucose transporter member 4 | *SLC2A4* |
| 66 | MOL000098 | Quercetin | Collagen alpha-1(III) chain | *COL3A1* |
| 67 | MOL000098 | Quercetin | DDB1- and CUL4-associated factor 5 | *DCAF5* |
| 68 | MOL000098 | Quercetin | Serine/threonine-protein kinase Chk2 | *CHEK2* |
| 69 | MOL000098 | Quercetin | Heat shock factor protein 1 | *HSF1* |
| 70 | MOL000098 | Quercetin | C-reactive protein | *CRP* |
| 71 | MOL000098 | Quercetin | Runt-related transcription factor 2 | *RUNX2* |
| 72 | MOL000098 | Quercetin | Ras association domain-containing protein 1 | *RASSF1* |
| 73 | MOL000098 | Quercetin | Cathepsin D | *CTSD* |
| 74 | MOL000098 | Quercetin | Insulin-like growth factor-binding protein 3 | *IGFBP3* |
| 75 | MOL000098 | Quercetin | Insulin-like growth factor II | *IGF2* |
| 76 | MOL000098 | Quercetin | Interferon regulatory factor 1 | *IRF1* |
| 77 | MOL000098 | Quercetin | Receptor tyrosine-protein kinase erbB-3 | *ERBB3* |
| 78 | MOL000098 | Quercetin | Serum paraoxonase/arylesterase 1 | *PON1* |
| 79 | MOL000098 | Quercetin | Type I iodothyronine deiodinase | *DIO1* |
| 80 | MOL000098 | Quercetin | Hexokinase-2 | *HK2* |
| 81 | MOL000098 | Quercetin | Ras GTPase-activating protein 1 | *RASA1* |
| 82 | MOL000098 | Quercetin | Glutathione S-transferase Mu 1 | *GSTM1* |
| 83 | MOL000098 | Quercetin | Glutathione S-transferase Mu 2 | *GSTM2* |
| 84 | MOL000953 | Cholesterol | Progesterone receptor | *PGR* |
| 85 | MOL000953 | Cholesterol | Mineralocorticoid receptor | *NR3C2* |
| 86 | MOL000953 | Cholesterol | Nuclear receptor coactivator 2 | *NCOA2* |
| 87 | MOL002773 | beta-Carotene | Vascular endothelial growth factor A | *VEGFA* |
| 88 | MOL002773 | beta-Carotene | Apoptosis regulator Bcl-2 | *BCL2* |
| 89 | MOL002773 | beta-Carotene | Caspase-9 | *CASP9* |
| 90 | MOL002773 | beta-Carotene | Caspase-3 | *CASP3* |
| 91 | MOL002773 | beta-Carotene | Caspase-8 | *CASP8* |
| 92 | MOL002773 | beta-Carotene | Cytochrome P450 3A4 | *CYP3A4* |
| 93 | MOL002773 | beta-Carotene | Serum albumin | *ALB* |
| 94 | MOL002773 | beta-Carotene | Caveolin-1 | *CAV1* |
| 95 | MOL002773 | beta-Carotene | Catenin beta-1 | *CTNNB1* |
| 96 | MOL002773 | beta-Carotene | Myc proto-oncogene protein | *MYC* |
| 97 | MOL002773 | beta-Carotene | Caspase-7 | *CASP7* |
| 98 | MOL000359 | Sitosterol | Progesterone receptor | *PGR* |
| 99 | MOL000359 | Sitosterol | Nuclear receptor coactivator 2 | *NCOA2* |
| 100 | MOL000359 | Sitosterol | Mineralocorticoid receptor | *NR3C2* |

**Table A3** Gene ontology (GO) terms of therapeutic target genes and their corresponding counts, *p-*value, and FDR. Through the GO enrichment of the key targets, 86 remarkably enriched (*p-*value ≤ 0.01) GO terms were obtained, indicating that some targets were involved in tumorigenesis.

| GO ID | Term | Count | *p*-value | FDR |
| --- | --- | --- | --- | --- |
| GO:0046982 | Protein heterodimerization activity | 14 | 4.41E-08 | 6.73E-06 |
| GO:0048037 | Cofactor binding | 13 | 7.74E-08 | 6.73E-06 |
| GO:0000987 | Proximal promoter sequence-specific DNA binding | 13 | 1.11E-07 | 6.73E-06 |
| GO:0001228 | DNA-binding transcription activator activity, RNA polymerase II-specific activity | 12 | 1.75E-07 | 7.94E-06 |
| GO:0033613 | Activating transcription factor binding | 6 | 4.36E-07 | 1.35E-05 |
| GO:0016209 | Antioxidant activity | 6 | 4.78E-07 | 1.35E-05 |
| GO:0000978 | RNA polymerase II proximal promoter sequence-specific DNA binding | 12 | 5.24E-07 | 1.35E-05 |
| GO:0044389 | Ubiquitin-like protein ligase binding | 10 | 6.41E-07 | 1.45E-05 |
| GO:0031625 | Ubiquitin protein ligase binding | 9 | 3.59E-06 | 7.22E-05 |
| GO:0003682 | Chromatin binding | 11 | 4.22E-06 | 7.64E-05 |
| GO:0019825 | Oxygen binding | 4 | 6.35E-06 | 1.05E-04 |
| GO:0020037 | Heme binding | 6 | 8.53E-06 | 1.29E-04 |
| GO:0001085 | RNA polymerase II transcription factor binding | 6 | 1.23E-05 | 1.59E-04 |
| GO:0005178 | Integrin binding | 6 | 1.23E-05 | 1.59E-04 |
| GO:0046906 | Tetrapyrrole binding | 6 | 1.43E-05 | 1.63E-04 |
| GO:0043295 | Glutathione binding | 3 | 1.44E-05 | 1.63E-04 |
| GO:1900750 | Oligopeptide binding | 3 | 1.92E-05 | 2.04E-04 |
| GO:0004601 | Peroxidase activity | 4 | 2.31E-05 | 2.32E-04 |
| GO:0051879 | Hsp90 protein binding | 4 | 2.57E-05 | 2.45E-04 |
| GO:0016684 | Oxidoreductase activity with peroxide as the acceptor | 4 | 3.16E-05 | 2.86E-04 |
| GO:0016705 | Oxidoreductase activity on paired donors, with incorporation or reduction of molecular oxygen | 6 | 3.68E-05 | 3.06E-04 |
| GO:0005496 | Steroid binding | 5 | 3.72E-05 | 3.06E-04 |
| GO:0035257 | Nuclear hormone receptor binding | 6 | 5.45E-05 | 4.01E-04 |
| GO:0004879 | Nuclear receptor activity | 4 | 5.53E-05 | 4.01E-04 |
| GO:0098531 | Transcription factor activity, direct ligand regulated sequence-specific DNA binding | 4 | 5.53E-05 | 4.01E-04 |
| GO:0019207 | Kinase regulator activity | 6 | 7.07E-05 | 4.77E-04 |
| GO:0070888 | E-box binding | 4 | 7.11E-05 | 4.77E-04 |
| GO:0003707 | Steroid hormone receptor activity | 4 | 1.04E-04 | 6.75E-04 |
| GO:0008047 | Enzyme activator activity | 9 | 1.14E-04 | 7.12E-04 |
| GO:0004197 | Cysteine-type endopeptidase activity | 4 | 1.20E-04 | 7.12E-04 |
| GO:0051427 | Hormone receptor binding | 6 | 1.22E-04 | 7.12E-04 |
| GO:0004364 | Glutathione transferase activity | 3 | 1.30E-04 | 7.36E-04 |
| GO:0070491 | Repressing transcription factor binding | 4 | 1.48E-04 | 7.94E-04 |
| GO:0016712 | Oxidoreductase activity on paired donors, with the incorporation or reduction of molecular oxygen, reduced flavin or flavoprotein as one donor, and incorporation of one atom of oxygen | 3 | 1.49E-04 | 7.94E-04 |
| GO:0033218 | Amide binding | 7 | 2.03E-04 | 1.05E-03 |
| GO:0072341 | Modified amino acid binding | 4 | 2.15E-04 | 1.08E-03 |
| GO:0046332 | SMAD binding | 4 | 3.19E-04 | 1.56E-03 |
| GO:0019903 | Protein phosphatase binding | 5 | 3.33E-04 | 1.59E-03 |
| GO:0019887 | Protein kinase regulator activity | 5 | 3.82E-04 | 1.77E-03 |
| GO:0042277 | Peptide binding | 6 | 4.33E-04 | 1.96E-03 |
| GO:0016248 | Channel inhibitor activity | 3 | 5.76E-04 | 2.54E-03 |
| GO:0001102 | RNA polymerase II activating transcription factor binding | 3 | 6.25E-04 | 2.64E-03 |
| GO:0004497 | Monooxygenase activity | 4 | 6.28E-04 | 2.64E-03 |
| GO:0004175 | Endopeptidase activity | 7 | 6.40E-04 | 2.64E-03 |
| GO:0001221 | Transcription cofactor binding | 3 | 6.76E-04 | 2.70E-03 |
| GO:0035258 | Steroid hormone receptor binding | 4 | 6.85E-04 | 2.70E-03 |
| GO:0030331 | Estrogen receptor binding | 3 | 8.46E-04 | 3.24E-03 |
| GO:0001161 | Intronic transcription regulatory region sequence-specific DNA binding | 2 | 8.93E-04 | 3.24E-03 |
| GO:0044213 | Intronic transcription regulatory region DNA binding | 2 | 8.93E-04 | 3.24E-03 |
| GO:0050839 | Cell adhesion molecule binding | 8 | 8.95E-04 | 3.24E-03 |
| GO:0042826 | Histone deacetylase binding | 4 | 1.19E-03 | 4.22E-03 |
| GO:0019902 | Phosphatase binding | 5 | 1.26E-03 | 4.38E-03 |
| GO:0004953 | Eicosanoid receptor activity | 2 | 1.30E-03 | 4.45E-03 |
| GO:0016765 | Transferase activity, transferring alkyl or aryl (other than methyl) groups | 3 | 1.42E-03 | 4.78E-03 |
| GO:0031072 | Heat shock protein binding | 4 | 1.47E-03 | 4.84E-03 |
| GO:0050661 | NADP binding | 3 | 1.51E-03 | 4.88E-03 |
| GO:0043621 | Protein self-association | 3 | 2.10E-03 | 6.66E-03 |
| GO:0019838 | Growth factor binding | 4 | 2.23E-03 | 6.96E-03 |
| GO:0004602 | Glutathione peroxidase activity | 2 | 2.34E-03 | 7.18E-03 |
| GO:0005123 | Death receptor binding | 2 | 2.65E-03 | 7.98E-03 |
| GO:0001091 | RNA polymerase II basal transcription factor binding | 2 | 2.97E-03 | 8.66E-03 |
| GO:0016538 | Cyclin-dependent protein serine/threonine kinase regulator activity | 2 | 2.97E-03 | 8.66E-03 |
| GO:0008395 | Steroid hydroxylase activity | 2 | 3.31E-03 | 9.50E-03 |
| GO:0030295 | Protein kinase activator activity | 3 | 3.64E-03 | 1.02E-02 |
| GO:0004709 | MAP kinase kinase kinase activity | 2 | 3.66E-03 | 1.02E-02 |
| GO:0001223 | Transcription coactivator binding | 2 | 4.04E-03 | 1.09E-02 |
| GO:0070330 | Aromatase activity | 2 | 4.04E-03 | 1.09E-02 |
| GO:0005506 | Iron ion binding | 4 | 4.17E-03 | 1.11E-02 |
| GO:0070001 | Aspartic-type peptidase activity | 2 | 4.43E-03 | 1.16E-02 |
| GO:0008234 | Cysteine-type peptidase activity | 4 | 4.59E-03 | 1.18E-02 |
| GO:0019209 | Kinase activator activity | 3 | 4.62E-03 | 1.18E-02 |
| GO:0070412 | R-SMAD binding | 2 | 4.83E-03 | 1.22E-02 |
| GO:0051219 | Phosphoprotein binding | 3 | 4.98E-03 | 1.24E-02 |
| GO:0051117 | ATPase binding | 3 | 5.17E-03 | 1.26E-02 |
| GO:0050662 | Coenzyme binding | 5 | 6.24E-03 | 1.51E-02 |
| GO:0017171 | Serine hydrolase activity | 4 | 6.45E-03 | 1.54E-02 |
| GO:0051213 | Dioxygenase activity | 3 | 6.80E-03 | 1.60E-02 |
| GO:0005504 | Fatty acid binding | 2 | 7.12E-03 | 1.65E-02 |
| GO:0042805 | Actinin binding | 2 | 7.62E-03 | 1.73E-02 |
| GO:0051059 | NF-kappaB binding | 2 | 7.62E-03 | 1.73E-02 |
| GO:0030291 | Protein serine/threonine kinase inhibitor activity | 2 | 8.14E-03 | 1.82E-02 |
| GO:0031406 | Carboxylic acid binding | 4 | 8.41E-03 | 1.86E-02 |
| GO:0097718 | Disordered domain specific binding | 2 | 8.68E-03 | 1.88E-02 |
| GO:0043177 | Organic acid binding | 4 | 8.74E-03 | 1.88E-02 |
| GO:0016651 | Oxidoreductase activity on NAD(P)H | 3 | 9.49E-03 | 2.02E-02 |
| GO:0016709 | Oxidoreductase activity on paired donors, with the incorporation or reduction of molecular oxygen, NAD(P)H as one donor, and the incorporation of one atom of oxygen | 2 | 9.80E-03 | 2.06E-02 |

**Table A4** Kyoto Encyclopedia of Genes and Genomes (KEGG) pathway of therapeutic target genes and their corresponding counts, *p*-value, and FDR. Through KEGG enrichment of the key targets, 89 remarkably enriched (*p-*value ≤ 0.01) pathways were obtained, indicating that numerous targets are involved in the processes of tumorigenesis and tumor progression.

| Term | Pathway | Count | *p*-value | FDR |
| --- | --- | --- | --- | --- |
| hsa05215 | Prostate cancer | 14 | 1.46E-13 | 1.45E-11 |
| hsa05418 | Fluid shear stress and atherosclerosis | 15 | 1.45E-12 | 7.17E-11 |
| hsa05167 | Kaposi sarcoma-associated herpesvirus infection | 16 | 8.13E-12 | 2.68E-10 |
| hsa05163 | Human cytomegalovirus infection | 17 | 1.34E-11 | 3.32E-10 |
| hsa05225 | Hepatocellular carcinoma | 15 | 2.33E-11 | 4.39E-10 |
| hsa05205 | Proteoglycans in cancer | 16 | 2.66E-11 | 4.39E-10 |
| hsa04933 | AGE-RAGE signaling pathway in diabetic complications | 12 | 8.81E-11 | 1.25E-09 |
| hsa05161 | Hepatitis B | 14 | 1.97E-10 | 2.44E-09 |
| hsa05210 | Colorectal cancer | 11 | 2.84E-10 | 3.12E-09 |
| hsa01524 | Platinum-based drug resistance | 10 | 9.79E-10 | 9.69E-09 |
| hsa04066 | HIF-1 signaling pathway | 11 | 1.49E-09 | 1.34E-08 |
| hsa05219 | Bladder cancer | 8 | 2.78E-09 | 2.29E-08 |
| hsa04210 | Apoptosis | 12 | 3.28E-09 | 2.49E-08 |
| hsa04668 | TNF signaling pathway | 11 | 4.16E-09 | 2.94E-08 |
| hsa05212 | Pancreatic cancer | 9 | 2.37E-08 | 1.56E-07 |
| hsa05134 | Legionellosis | 8 | 3.19E-08 | 1.98E-07 |
| hsa01521 | EGFR tyrosine kinase inhibitor resistance | 9 | 3.77E-08 | 2.20E-07 |
| hsa05213 | Endometrial cancer | 8 | 4.92E-08 | 2.70E-07 |
| hsa05206 | MicroRNAs in cancer | 15 | 6.98E-08 | 3.64E-07 |
| hsa04919 | Thyroid hormone signaling pathway | 10 | 1.20E-07 | 5.92E-07 |
| hsa05223 | Non-small cell lung cancer | 8 | 1.39E-07 | 6.54E-07 |
| hsa05160 | Hepatitis C | 11 | 1.52E-07 | 6.82E-07 |
| hsa05169 | Epstein-Barr virus infection | 12 | 2.61E-07 | 1.12E-06 |
| hsa04115 | p53 Signaling pathway | 8 | 2.77E-07 | 1.14E-06 |
| hsa04010 | MAPK signaling pathway | 14 | 4.02E-07 | 1.59E-06 |
| hsa04215 | Apoptosis induced by multiple species | 6 | 4.71E-07 | 1.79E-06 |
| hsa04151 | PI3K-Akt signaling pathway | 15 | 6.27E-07 | 2.30E-06 |
| hsa05224 | Breast cancer | 10 | 8.68E-07 | 3.07E-06 |
| hsa04012 | ErbB signaling pathway | 8 | 1.01E-06 | 3.45E-06 |
| hsa04218 | Cellular senescence | 10 | 1.89E-06 | 6.23E-06 |
| hsa05230 | Central carbon metabolism in cancer | 7 | 2.01E-06 | 6.24E-06 |
| hsa05222 | Small cell lung cancer | 8 | 2.02E-06 | 6.24E-06 |
| hsa04926 | Relaxin signaling pathway | 9 | 2.77E-06 | 8.11E-06 |
| hsa05164 | Influenza A | 10 | 2.79E-06 | 8.11E-06 |
| hsa01522 | Endocrine resistance | 8 | 3.00E-06 | 8.49E-06 |
| hsa05170 | Human immunodeficiency virus 1 infection | 11 | 3.45E-06 | 9.49E-06 |
| hsa05162 | Measles | 9 | 4.54E-06 | 1.21E-05 |
| hsa05226 | Gastric cancer | 9 | 8.52E-06 | 2.22E-05 |
| hsa04510 | Focal adhesion | 10 | 1.33E-05 | 3.37E-05 |
| hsa04370 | VEGF signaling pathway | 6 | 1.58E-05 | 3.82E-05 |
| hsa05416 | Viral myocarditis | 6 | 1.58E-05 | 3.82E-05 |
| hsa05235 | PD-L1 expression and PD-1 checkpoint pathway in cancer | 7 | 1.66E-05 | 3.92E-05 |
| hsa05143 | African trypanosomiasis | 5 | 2.07E-05 | 4.77E-05 |
| hsa05166 | Human T-cell leukemia virus 1 infection | 10 | 3.05E-05 | 6.86E-05 |
| hsa04064 | NF-kappa B signaling pathway | 7 | 3.56E-05 | 7.84E-05 |
| hsa04915 | Estrogen signaling pathway | 8 | 3.77E-05 | 8.11E-05 |
| hsa04659 | Th17 cell differentiation | 7 | 5.51E-05 | 1.16E-04 |
| hsa05214 | Glioma | 6 | 6.30E-05 | 1.30E-04 |
| hsa00980 | Metabolism of xenobiotics by cytochrome P450 | 6 | 6.79E-05 | 1.32E-04 |
| hsa05133 | Pertussis | 6 | 6.79E-05 | 1.32E-04 |
| hsa05220 | Chronic myeloid leukemia | 6 | 6.79E-05 | 1.32E-04 |
| hsa05145 | Toxoplasmosis | 7 | 7.81E-05 | 1.49E-04 |
| hsa05204 | Chemical carcinogenesis | 6 | 1.04E-04 | 1.94E-04 |
| hsa05165 | Human papillomavirus infection | 11 | 2.07E-04 | 3.76E-04 |
| hsa04657 | IL-17 signaling pathway | 6 | 2.09E-04 | 3.76E-04 |
| hsa05152 | Tuberculosis | 8 | 2.34E-04 | 4.08E-04 |
| hsa05146 | Amoebiasis | 6 | 2.35E-04 | 4.08E-04 |
| hsa04910 | Insulin signaling pathway | 7 | 2.61E-04 | 4.45E-04 |
| hsa04014 | Ras signaling pathway | 9 | 2.71E-04 | 4.55E-04 |
| hsa05231 | Choline metabolism in cancer | 6 | 2.94E-04 | 4.85E-04 |
| hsa05221 | Acute myeloid leukemia | 5 | 3.45E-04 | 5.60E-04 |
| hsa05216 | Thyroid cancer | 4 | 3.58E-04 | 5.71E-04 |
| hsa04625 | C-type lectin receptor signaling pathway | 6 | 3.84E-04 | 6.03E-04 |
| hsa04928 | Parathyroid hormone synthesis, secretion and action | 6 | 4.26E-04 | 6.58E-04 |
| hsa04917 | Prolactin signaling pathway | 5 | 4.54E-04 | 6.91E-04 |
| hsa04931 | Insulin resistance | 6 | 4.70E-04 | 7.05E-04 |
| hsa04921 | Oxytocin signaling pathway | 7 | 5.11E-04 | 7.54E-04 |
| hsa04630 | JAK-STAT signaling pathway | 7 | 7.19E-04 | 1.05E-03 |
| hsa04071 | Sphingolipid signaling pathway | 6 | 7.87E-04 | 1.13E-03 |
| hsa04152 | AMPK signaling pathway | 6 | 8.23E-04 | 1.16E-03 |
| hsa05144 | Malaria | 4 | 1.06E-03 | 1.47E-03 |
| hsa04068 | FoxO signaling pathway | 6 | 1.35E-03 | 1.86E-03 |
| hsa05202 | Transcriptional misregulation in cancer | 7 | 1.61E-03 | 2.19E-03 |
| hsa05142 | Chagas disease (American trypanosomiasis) | 5 | 2.59E-03 | 3.47E-03 |
| hsa04620 | Toll-like receptor signaling pathway | 5 | 2.70E-03 | 3.57E-03 |
| hsa01523 | Antifolate resistance | 3 | 2.86E-03 | 3.72E-03 |
| hsa04920 | Adipocytokine signaling pathway | 4 | 3.75E-03 | 4.81E-03 |
| hsa05120 | Epithelial cell signaling in Helicobacter pylori infection | 4 | 3.95E-03 | 5.00E-03 |
| hsa04726 | Serotonergic synapse | 5 | 4.17E-03 | 5.22E-03 |
| hsa00982 | Drug metabolism - cytochrome P450 | 4 | 4.37E-03 | 5.33E-03 |
| hsa05218 | Melanoma | 4 | 4.37E-03 | 5.33E-03 |
| hsa05140 | Leishmaniasis | 4 | 4.82E-03 | 5.81E-03 |
| hsa04110 | Cell cycle | 5 | 5.74E-03 | 6.84E-03 |
| hsa00983 | Drug metabolism - other enzymes | 4 | 6.07E-03 | 7.15E-03 |
| hsa04140 | Autophagy - animal | 5 | 6.55E-03 | 7.63E-03 |
| hsa04662 | B cell receptor signaling pathway | 4 | 6.92E-03 | 7.97E-03 |
| hsa04930 | Type II diabetes mellitus | 3 | 8.75E-03 | 9.89E-03 |
| hsa04020 | Calcium signaling pathway | 6 | 8.80E-03 | 9.89E-03 |
| hsa05323 | Rheumatoid arthritis | 4 | 9.94E-03 | 1.11E-02 |
